# Mn^4+^-Doped Magnesium Titanate—A Promising Phosphor for Self-Referenced Optical Temperature Sensing

**DOI:** 10.3390/s18020668

**Published:** 2018-02-24

**Authors:** Francesca Venturini, Michael Baumgartner, Sergey M. Borisov

**Affiliations:** 1 Institute of Applied Mathematics and Physics, Zurich University of Applied Sciences, Winterthur 8401, Switzerland; bamm@zhaw.ch; 2 Institute of Analytical Chemistry and Food Chemistry, Graz University of Technology, Stremayrgasse 9, 8010 Graz, Austria

**Keywords:** phosphor, optical sensor, temperature sensor, manganese, luminescence

## Abstract

Phosphors based on magnesium titanate activated with Mn${}^{4+}$ ions are of great interest because, when excited with blue light, they display a strong red-emitting luminescence. They are characterized by a luminescence decay which is strongly temperature dependent in the range from −50 ${}^{\circ }$C to 150 ${}^{\circ }$C, making these materials very promising for temperature sensing in the biochemical field. In this work, the optical and thermal properties of the luminescence of Mg${}_{2}$TiO${}_{4}$ are investigated for different Mn${}^{4+}$ doping concentrations. The potential of this material for temperature sensing is demonstrated by fabricating a fiber optic temperature microsensor and by comparing its performance against a standard resistance thermometer. The response of the fiber optic sensor is exceptionally fast, with a response time below 1 s in the liquid phase and below 1.1 s in the gas phase.

## 1. Introduction

Temperature is a parameter frequently measured in many applications which include, among others, biosensing, environmental monitoring, medicine and diagnostics. Among the many sensing schemes to measure temperature, fiber optic sensors have attracted much interest because they offer several advantages: small sensor size, and consequently fast response time, accuracy, electromagnetic immunity, non-contact and in-situ capabilities [1,2].

One of the newest approaches to optical fiber sensing is the “Lab-on-Fiber” (LOF) one, which consists in integrating onto optical fibers advanced functional materials at micro- and nano- scales [3]. Among the LOF technology platforms, “Lab-on-Tip” devices are particularly attractive because of the simplicity of the integration of the sensing material on the fiber tip [4]. The application of LOF approach to temperature sensing has been demonstrated using several measuring principles, like for example Fabry-Perot interferometers [5,6], Fiber Bragg gratings [7,8] or surface plasmon resonance [9,10]. Although often moderate sensitivities are reported, with resolutions of 0.55 ${}^{\circ }$C [5], the response time may be several seconds [5,8].

One of the most versatile sensing schemes for optical temperature measurement is luminescence detection, which has therefore been widely investigated in the past years. Since luminescence emission is a temperature dependent process, several classes of luminescent materials have been proposed depending on the temperature range, on the sensitivity and on the stability required for the application. Some examples, to cite only a few, are inorganic phosphors such as manganese(IV)-activated magnesium fluorogermanate [2,11], ruby [12], alexandrite [13], europium(III)-doped yttrium oxide and other rare-earth phosphors [14,15,16,17], metal-organic complexes like ruthenium(II) polypyridyls [18,19], and europium(III) complexes [20,21,22]. A comprehensive review can be found in [23].

More recently, phosphors characterized by Mn${}^{4+}$ ions incorporated in different hosts have attracted renewed interest because of their spectral properties. In fact, the activation with Mn${}^{4+}$ ions results in a strong red-emitting luminescence which is excited by blue light. For this reason, Mn${}^{4+}$ is an attractive substitute for well-established rare-earth activators in red phosphors [24,25,26,27,28,29].

Mn${}^{4+}$-activated magnesium fluoromanganate was originally developed as a red-emitting component of fluorescent lamps and is today used to improve the spectral emission of white LEDs. Therefore, a weak temperature quenching is desired to keep the brightness and chromatic properties of white light with increasing temperatures [30,31]. On the other hand, a temperature dependent emission-changing characteristic enables these phosphors to be used for thermographic sensing. For these applications, the temperature dependency of luminescence lifetime is particularly attractive due to self-reference character of this parameter and simplicity of required instrumentation. Notably, Mn(IV)-activated magnesium fluorogermanate is a promising material for high-temperature phosphor thermometry [31] but it does not provide adequate resolution at ambient conditions with a sensitivity of the decay time about 0.2%/K [32]).

In this work, to achieve a fast response time and a good resolution, a new type of LOF luminescence-based sensor was developed, which is insensitive against external factors like pressure and fluctuations of the intensity of the light source. The luminescence emission Mn${}^{4+}$-activated magnesium titanate was investigated as function of temperature and different doping concentrations. The results show that a good temperature sensitivity, between −0.5%/K and −0.8%/K, can be reached with all Mn${}^{4+}$ concentrations, for temperatures from 0 ${}^{\circ }$C to 80 ${}^{\circ }$C. The material was then used to fabricate a fiber optic microsensor whose performance was investigated. The results show that the microsensor has a robust, reproducible and very fast response. This demonstrates the potential of this material for thermographic applications in the temperature range studied.

## 2. Experimental

### 2.1. Synthesis of Mn${}^{4+}$-Doped Mg${}_{2}$TiO${}_{4}$ Crystals

The phosphors were prepared via sol-gel method from magnesium nitrate hexahydrate (5.128 g), manganese(II) nitrate tetrahydrate (0–0.0251 g) and titanium(IV) butoxide (3.403–3.369 g). Titanium(IV) butoxide is dissolved in acetic acid (10 mL) and added to the solution of magnesium nitrate and manganese(II) nitrate in ethanol (40 mL). 1 mL of water is added to promote hydrolysis. After stirring at room temperature for 2 h, the solvents are evaporated in a water bath and the residue is dried in an oven at 100 ${}^{\circ }$C overnight. The gel is calcinated at 500 ${}^{\circ }$C, homogenized in a mortar and sintered at 1100 ${}^{\circ }$C for 24 h. The resulting phosphors are homogenized in a ball mill to give a microcrystalline powder.

### 2.2. Instruments and Measurements for Phosphor Characterization

The characterization of the material was performed by investigating the emission properties of Mg${}_{2}$TiO${}_{4}$ samples with different Mn${}^{4+}$ doping concentrations as a function of temperature. To perform the measurements, the powder crystals were immobilized in semi-transparent thin films of two-component silicon rubber (RT 601 Elastosil, Wacker Chemie AG, Munich, Germany). High emission intensities were achieved by spin-coating three layers one over the other.

To control the temperature of the samples, these were placed in good thermal contact with a Cu-plate. The temperature of this plate was varied between 0 ${}^{\circ }$C and 80 ${}^{\circ }$C using a Peltier element and stabilized with a temperature controller (PTC10, Stanford Research Systems, Sunnyvale, CA, USA).

**Figure 1:**
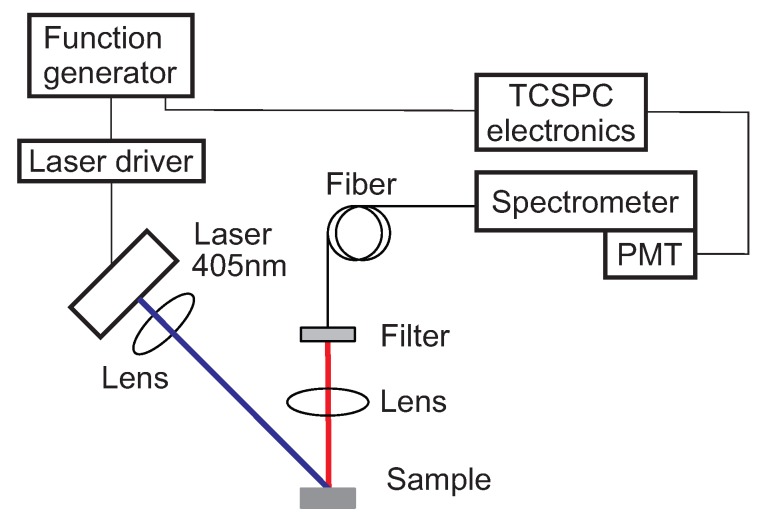
Scheme of the optical experimental setup. Blue is the excitation, red is the luminescence optical path. PMT: photomultiplier.

The optical setup used for the measurement of both the emission spectra and the decay time is shown schematically in Figure 1. The luminescence intensity decay was measured using time-correlated-single-photon-counting (TCSPC) technique. This allows for a very high resolution of the intensity decay curve.

The excitation light was provided by a 405 nm laser diode (DL-5146, SANYO Electric Co., Ltd., Tottori, Japan) focused on the surface of the samples with a collimation lens (C671TME-405 ThorLabs, Newton, NJ, USA). The luminescence was focused by a lens (Edmund Optics Inc., Karlsruhe, Germany) on a fiber bundle and then analyzed using a monochromator (Bruker 250 sm/is, Bruker Optics, Inc., The Woodlands, TX, USA) equipped with a photomultiplier (H10721-01, Hamamatsu Photonics K.K., Shimokanzo, Iwata City, Shizuoka Pref., 438-0193, Japan). To suppress excitation light reflected by the sample surface, the emission channel was equipped with an optical density OD5 long pass filter with cut-off at 594 nm (Semrock 594 LP Edge Basic long pass, Semrock, Inc., Rochester, NY, USA). The excitation pulse was generated by a frequency generator (Agilent 33220A, Agilent Technologies, Inc., Loveland, CO, USA) which sent the synchronization signal to the acquisition electronics (TimeHarp 260 NANO, PicoQuant GmbH, Berlin, Germany). The duration of the pulses was typically 10 μs, since the decay times to measure are between 200 and 600 μs. The duration of the pulses as well as the intensity of the laser were adjusted to maximize the signal-to-noise ratio, avoiding “pile-up” effects [33]. The instrument response function (IRF) was measured with a diffusing quartz plate and was established to be much shorter than the measured luminescence decay times. Therefore, no mathematical analysis of the convolution between the IRF and the real fluorescence signal was necessary.

For the measurement of the spectral properties, the spectrometer slits were adjusted to have a narrow band pass transmission of 2 nm and the range 600 nm to 800 nm was scanned. For the decay time measurement, the spectrometer was centered at ca. 660 nm where the maximum of the emission intensity was observed.

The measurement of the decay time in the larger temperature range (−50 ${}^{\circ }$C to 150 ${}^{\circ }$C) were performed by exciting the phosphor powder contained in a glass vial with a 465 nm LED, whose intensity was sinusoidally modulated at 300 Hz with a two-phase lock-in amplifier (SR830, Stanford Research Instruments Inc., Sunnyvale, CA, USA). The luminescence emission light was detected with a photomultiplier (H5701-02, Hamamatsu Photonics K.K., Shimokanzo, Iwata City, Shizuoka Pref., 438-0193, Japan) after passing a long pass filter (RG-630, Schott AG, Mainz, Germany). A bifurcated optical fiber bundle (LEONI Prinz Fiber Optics GmbH, Germany) was used to collect the excitation light to the phosphor and to guide back the emission. The vial was positioned into a cavity of a home-made aluminum block which was placed on a heating plate. The metal block was cooled down to about −50 ${}^{\circ }$C with liquid nitrogen and afterwards warmed with help of the heating plate to approximately 150 ${}^{\circ }$C. The temperature in the vial was measured by a standard PT-100 resistance thermometer.

### 2.3. Microsensor Fabrication and Test

The temperature microsensor was prepared by coating the tip of a 400 μm glass fiber (Industrial Fiber Optics, Tempe, AZ, USA) with a “cocktail” containing 100 mg temperature phosphor (0.40% doping) and 720 mg of 7% wt solution of Cytop M in a perfluorinated solvent (Asahi glass Co., Tokyo, Japan). After coating, the fiber was dried in an oven at 70 ${}^{\circ }$C overnight.

**Figure 2:**
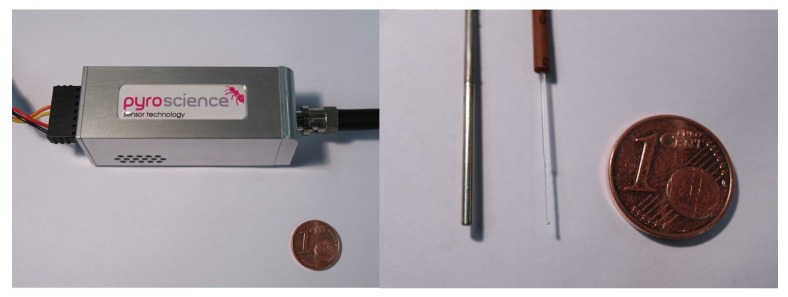
Left: Photographic images of the compact phase fluorometer from PyroScience. Right: from left to right: photographic image of resistance thermometer PT-100, fiber optic temperature microsensor and 1 euro cent for size comparison.

The microsensor was tested by using a compact phase fluorometer (Pyro Science GmbH, Aachen, Germany) (Figure 2). To ensure compatibility to the temperature phosphor, the compact fluorometer was modified with a 470 nm blue LED for the excitation, dichroic DT Cyan filter (Qioptiq Photonics GmbH & Co KG, Munich, Germany) which transmits the excitation light towards the optical fiber but reflects the emission light towards the photodetector, and the combination of Deep Golden Amber plastic filter (Lee filters, Burbank, CA, US) and OG 590 filter (Schott AG, Mainz, Germany) in front of the photodetector. The decay times were determined via phase-shift measurement in the frequency domain. The modulation frequency was 500 Hz. The temperature was controlled using a cryostat (Thermo Haake DC50, Thermo Fisher Scientific, Inc., Newington, NH, USA) and a resistance thermometer PT-100 supplied by Pyro Science GmbH.

## 3. Results and Discussion

### 3.1. Photoluminescence Spectra

The emission spectra were measured at temperatures between 4 ${}^{\circ }$C and 77 ${}^{\circ }$C for samples with different Mn${}^{4+}$ doping concentrations. For all the concentrations investigated, the spectrum showed similar features, with a narrow peak at approximately 660 nm and a broad shoulder from 630 nm to 720 nm. The narrow peak at 660 nm is due to the spin-forbidden transition from the lowest excited state ${}^{2}$E to the ground state ${}^{4}$A${}_{2}$ of the Mn${}^{4+}$ ion. The broad shoulder can be attributed to lattice vibration associated with the ${}^{2}$E →${}^{4}$A${}_{2}$ zero-phonon transition [34].

**Figure 3:**
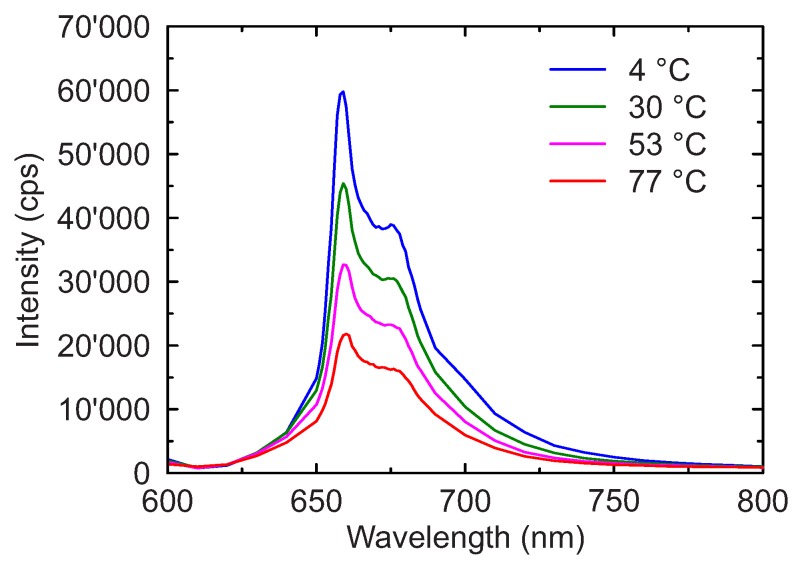
Emission spectrum of the sample with a Mn${}^{4+}$ doping concentration of 0.40% at different temperatures.

An example of the temperature dependence of the emission spectrum is shown in Figure 3 for a sample with a Mn${}^{4+}$ doping concentration of 0.40%.

It is evident that a temperature increase reduces the emission intensity across the entire spectrum and does not result into any significant change in the spectral features. Additionally, no measurable shift of the narrow peak at 660 nm was observed with increasing temperatures as reported for other Mn${}^{4+}$ compounds [31]. Consistently with what was previously reported for Mn${}^{4+}$ concentrations between 0% and 0.25% [27], the spectrum associated with the ${}^{2}$E → ${}^{4}$A${}_{2}$ transition maintains identical features up to 1% concentration.

### 3.2. Luminescence Quantum Yields

The absolute quantum yield for the phosphor powders determined with the integrating spheres from Horiba is shown in Table 1. For reference, the decay times discussed in detail in the next sections are also reported in the table.

As it can be seen, the quantum yield is very high for Mn${}^{4+}$ concentrations up to 0.10%. Further increase of Mn${}^{4+}$ results in a decrease of the quantum yield due to the concentration quenching effect [27]. Despite the lower quantum yield, the phosphor with 0.40% doping displays a greater brightness than that of the phosphor with 0.10% doping. This is due to the four-fold more efficient absorption of the former. This aspect is important for the design of the temperature optodes since the luminescence brightness and not the quantum yield determines the signal to noise ratio.

### 3.3. Luminescence Lifetimes

**Figure 4:**
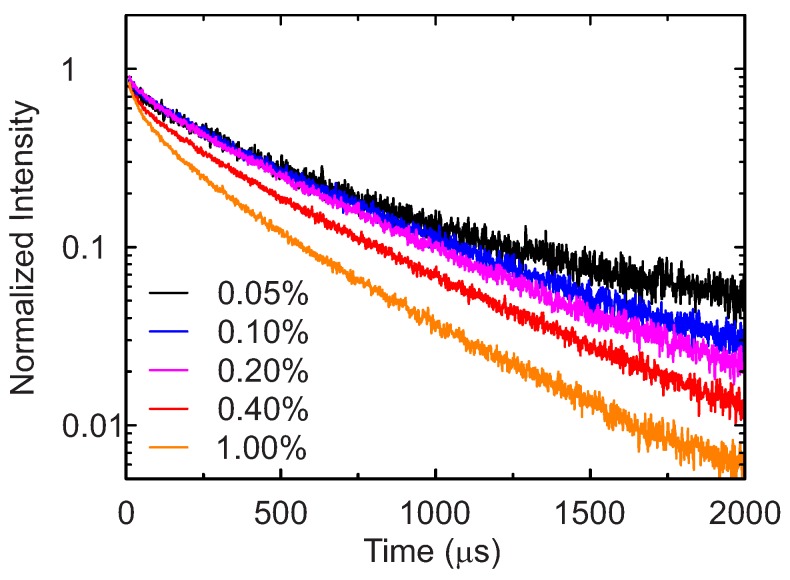
Normalized luminescence intensity decay at 20 ${}^{\circ }$C for samples with different doping concentrations.

The luminescence intensity decays for the phosphors with different doping levels measured at 20${}^{\circ }$C are shown in Figure 4. The intensities are normalized to better display the changes with the doping. The decay time decreases with increasing Mn${}^{4+}$-concentration, which is in good agreement with the quenching effect observed for the luminescence quantum yields.

**Figure 5:**
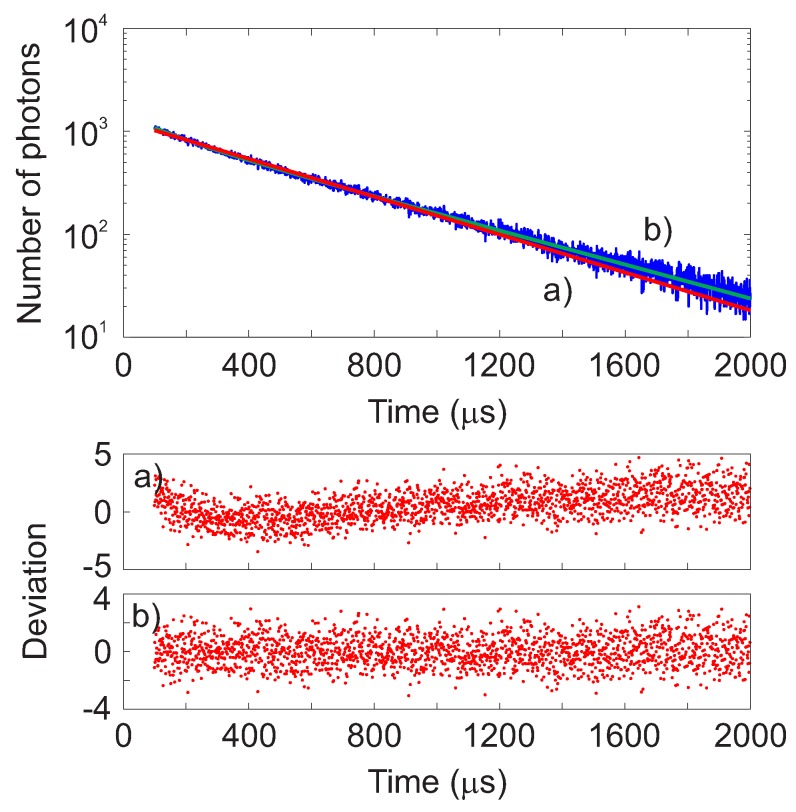
Upper panel: Time-domain intensity decay time at 20 ${}^{\circ }$C for the sample with 0.20% doping. The red solid line (**a**) shows the monoexponential function fit with $\chi_{R}^{2}$ = 1.97. The red green line (**b**) shows the double exponential function fit with $\chi_{R}^{2}$ = 1.23. Lower panels: deviation plots for the mono- (**a**) and double (**b**) exponential fits.

To determine the decay time, the luminescence intensity curves were fitted using both a monoexponential and a double-exponential decay function. In the upper panel of Figure 5, the time-domain intensity decay at 20 ${}^{\circ }$C for the sample with 0.20% doping is shown as an example. The data were fitted using monoexponential decay response (Figure 5a, red line) and a double-exponential decay response (Figure 5b, green line). To estimate quantitatively the goodness of the fit, a nonlinear least squares analysis was performed and the reduced $\chi_{R}^{2}$ calculated for both cases. For each intensity curve, the deviations were calculated as the difference between the measured and the calculated data, weighted according to the standard deviation of each data point [33]. These are shown in Figure 5 in the lower panels for both the mono- and double- exponential fit functions.

The results indicate that the intensity decay can be reasonably well approximated by a monoexponential behavior, consistently with previous observations [28]. There are indications of a double-exponential decay behavior, as indicated by the value of $\chi_{R}^{2}$. The highest deviations were observed for the sample with the highest doping concentration (1.00%) at the temperature of 77 ${}^{\circ }$C, where the $\chi_{R}^{2}$ is as high as 6.2. The presence of a faster decay component was reported in K${}_{2}$SiF${}_{6}$:Mn${}^{4+}$ and attributed to thermal activation of defect centers [35]. However, for practical applications, it is enough to characterize the intensity decay of a sample in terms of a single apparent or mean decay time. For this reason, in this work the data where analyzed using a monoexponential fitting function.

**Figure 6:**
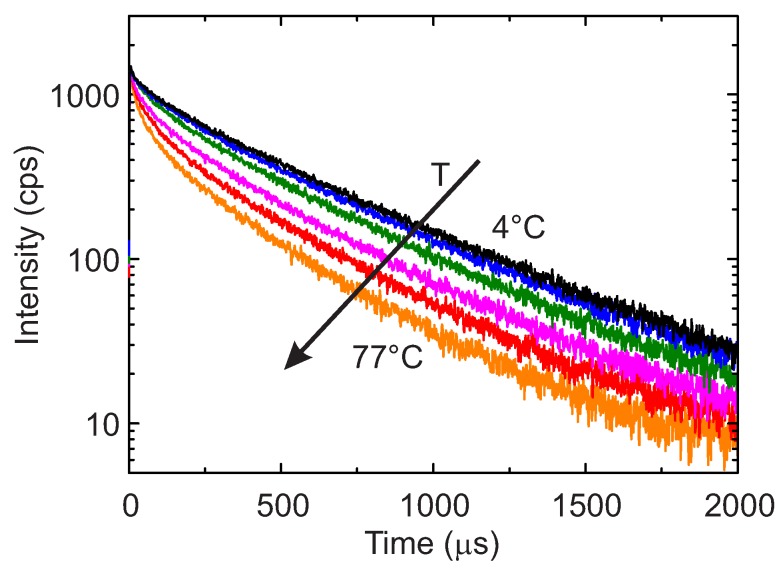
Luminescence intensity decay of the sample with a Mn${}^{4+}$ doping concentration of 0.40% for temperatures the 4 ${}^{\circ }$C, 12 ${}^{\circ }$C, 29 ${}^{\circ }$C, 45 ${}^{\circ }$C, 61 ${}^{\circ }$C and 77 ${}^{\circ }$C. The direction of the increasing temperatures is also shown.

The typical temperature dependence of the luminescence intensity decay is shown in Figure 6 for a sample with a Mn${}^{4+}$ doping concentration of 0.40% for six temperatures between 4 ${}^{\circ }$C and 77 ${}^{\circ }$C. A visible decrease of the decay time is observed as the temperature increases. It is also evident that as the temperature increases, the decay curves show higher deviation from the monoexponential profile.

**Figure 7:**
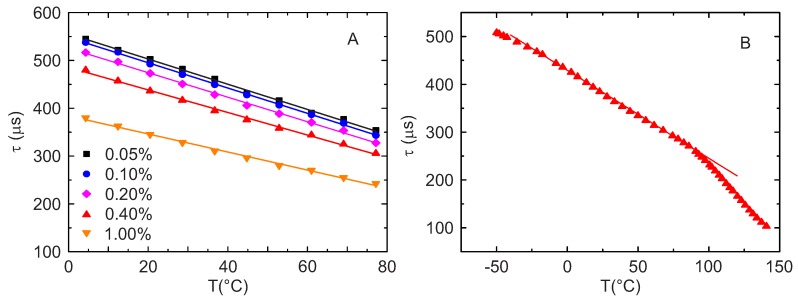
(**A**) Temperature dependence of the monoexponential lifetime for samples with different Mn${}^{4+}$ doping concentrations; (**B**) Temperature dependence of a sample with 0.40% doping concentration in a broader temperature range obtained in the frequency domain. Points: Measurements, solid lines: linear fit. In B, the linear fit is performed in the temperature range from −30 ${}^{\circ }$C to 80 ${}^{\circ }$C.

The decay times in dependence of the temperature for different doping concentrations in the range between 0 ${}^{\circ }$C and 80 ${}^{\circ }$C are summarized in Figure 7, A. In this range, the temperature dependence of the decay time can be well described by a linear behavior, also shown in Figure 7, A for all the doping concentrations. The coefficient of determination R${}^{2}$ was between 0.995 and 0.999. In Figure 7, B a sample with a Mn${}^{4+}$ doping concentration of 0.40% was investigated in a broader temperature range, between −50 ${}^{\circ }$C and 150 ${}^{\circ }$C. As can be seen, the material is usable in the broader temperature range, since it maintains a strong temperature dependence. Although the calibration function becomes more complex in this broader temperature range, the dependency in the range from −30 ${}^{\circ }$C to 80 ${}^{\circ }$C still obeys linear behavior (R${}^{2}=$ 0.996) making the sensor convenient to calibrate and use in this physiologically relevant temperature range.

**Figure 8:**
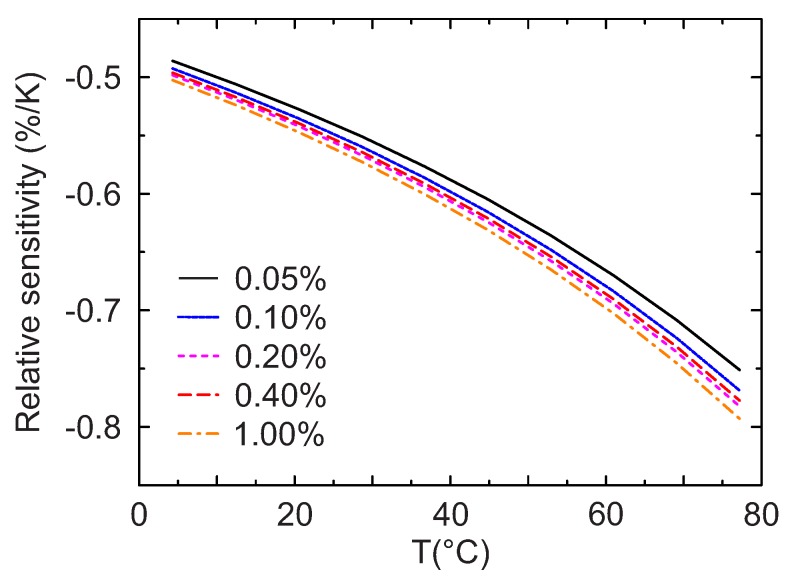
Temperature dependence of the relative sensitivity *s* for different Mn${}^{4+}$ doping concentrations.

The relative temperature sensitivity *s* defined as [1]

$$s=\frac{\Delta \tau /\tau }{\Delta T}$$

is shown in Figure 8, where τ is the observed lifetime, Δτ and ΔT are the variations of the lifetime and temperature respectively. As can be seen from Figure 8, the relative sensitivity is similar for all doping levels, with values between −0.5%/K at low temperatures and −0.8%/K at approximately 80 ${}^{\circ }$C. These results show that for all the concentrations this material maintains a high sensitivity over the entire range of temperatures investigated.

The values obtained for Mn${}^{4+}$-activated magnesium titanate are thus significantly higher than those for Mn${}^{4+}$-activated magnesium fluorogermanate (about −0.2 %/K for the physiologically relevant temperatures) [32] and are comparable to those of the highly sensitive chromum(III)-activated yttrium aluminum borate (∼−0.7%/K for microcrystalline powder) [17,32]. This makes the new material well suited for temperature measurements in biotechnology and environmental analysis as well as for temperature compensation of other optical sensors (oxygen, pH, etc.) operating in the same conditions.

### 3.4. Fiber Optic Microsensor

The fiber optic microsensor was manufactured by dispersing the phosphor microparticles (0.40% doping) in a solution of a perfluorinated polymer and coating the obtained composition on the distal end of a quartz optical fiber. The diameter of the fiber was 400 μm but fibers with smaller diameters or tapered fibers can also be used. Nevertheless, even in this design the sensor is much smaller than a standard resistance thermometer (Figure 2).

**Figure 9:**
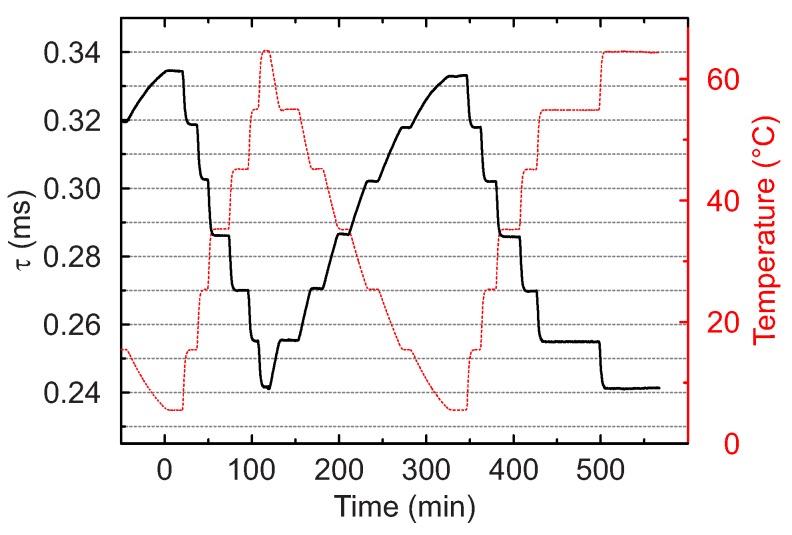
Response of the luminescence decay time of the fiber optic temperature microsensor (black trace). The temperature in the calibration chamber is measured by PT-100 resistance thermometer (red trace).

Figure 9 shows the response of the temperature microsensor. As can be seen, the response is highly reproducible: neither hysteresis nor drift was observed during the measurement cycle of over 10 h.

**Figure 10:**
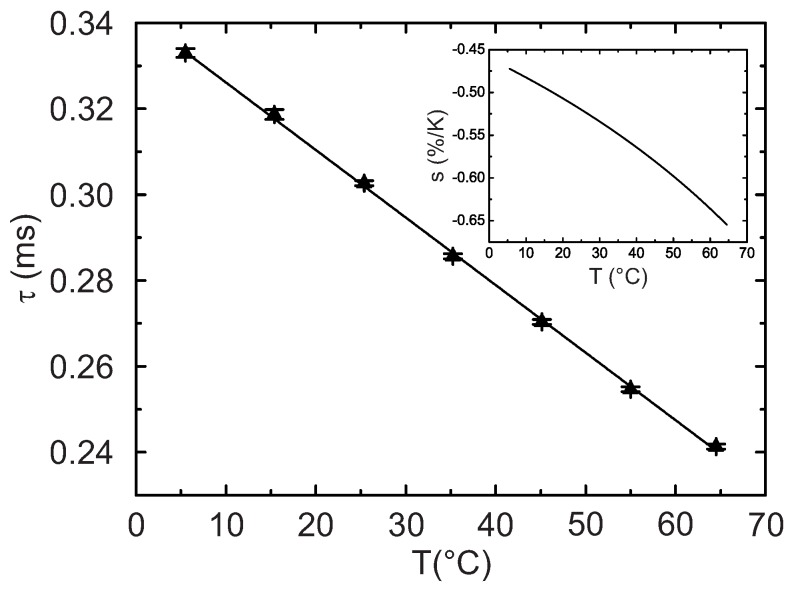
Temperature dependency of the luminescence decay time for the fiber optic temperature microsensor. The insert shows the relative sensitivity.

The Arrhenius plot (Figure 10) is similar but not completely identical to the one obtained in the time domain measurements (Figure 7). This can be explained by the fact that the phase-shift method allows accessing only the average decay times with the contributions from the faster and the slower-decaying components. Moreover, the longer decaying component is partially demodulated at the modulation frequencies used. Some contribution of background luminescence which results in lower measured phase shift (and consequently shorter decay time calculated) cannot be excluded. Nevertheless, the Arrhenius plot and the relative sensitivity plots are very similar in shape to those obtained in the time domain. In addition, the Arrhenius plot can be almost ideally fit with the linear equation (R${}^{2}$ = 0.9991).

**Figure 11:**
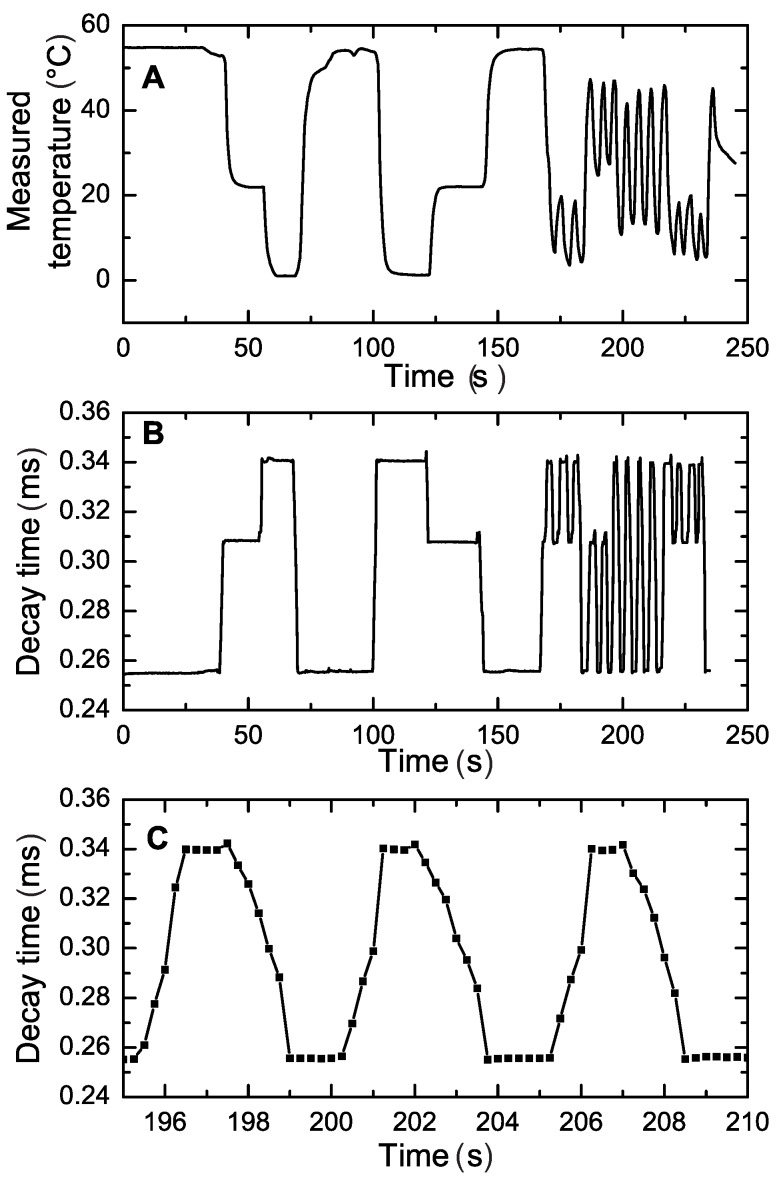
Dynamic response of the temperature resistance thermometer PT-100 (**A**) and fiber optic microsensor (**B**,**C**) to temperature changes between the beakers with water kept at 55 ${}^{\circ }$C, 22 ${}^{\circ }$C and ∼1.3 ${}^{\circ }$C (ice water). C is the zoom-in for the interval 195–210 s corresponding to the curve B.

Although optical temperature sensors can be easily realized in a variety of formats including planar optodes and spots, fiber optic microsensors, particularly, offer strong advantages over conventional analytical tools, such as resistance thermometers, in respect to the response times. In fact, their small size (Figure 2) allows for virtually instantaneous response. This advantage is clearly demonstrated in Figure 11. It is evident that the resistance thermometer can only resolve comparably slow temperature changes (time 0–150 s) but cannot follow the fast changes in temperature (Figure 11A) resulting in error as high as 10 ${}^{\circ }$C. On the contrary, the response of the fiber optical microsensor is much faster (Figure 11B). The response occurs within 1 s time (Figure 11C), however this time is likely to be overestimated due to time needed to transfer the sensor from one beaker to another. Moreover, sensors with smaller diameter are expected to be even faster.

For a quantitative comparison with other types of temperature sensors, the response time $t_{90}$, defined as the time needed to reach 90% of the final value, was found to be below 1 s in the liquid phase and 1.1 s in the gas phase, the latter measured for a temperature difference of 40 ${}^{\circ }$C and a gas flow of 5 m/s. Resistance thermometers faster than PT-100 with a $t_{90}$ in the liquid phase below 1 s are commercially available (see for example the P0K1.161.2I.B.050 by Innovative Sensor Technology IST AG, Ebnat-Kappel, Switzerland, or the 32205113 by TE Connectivity Company, Shrewsbury, MA, USA). However, in the gas phase even these models show a $t_{90}$ response time from 10 to 30 s in non-stagnant conditions (air flow of 1 m/s).

Finally, the resolution of the fiber optic microsensor was estimated based on its sensitivity and the phase noise of the device. The calculated resolution was 0.05 K at 25 ${}^{\circ }$C. This is a rough estimate, since the observed noise could also be caused by small temperature fluctuations. In addition, neither the hardware nor the settings of the read-out device were fully optimized for the current sensor, so it is expected that a significant improvement in resolution is possible.

## 4. Conclusions

The temperature dependent luminescence emission of Mn${}^{4+}$-doped Mg${}_{2}$TiO${}_{4}$ phosphor was investigated for different doping concentrations in the temperature range from −50 ${}^{\circ }$C to 150 ${}^{\circ }$C. The emission spectrum is characterized by a sharp peak at ca. 660 nm and a broad shoulder extending up to 720 nm, which decrease in intensity but do not change in shape significantly with increasing temperatures. The luminescent intensity decay is characterized by an almost monoexponential decay; the decay time decreases with increasing doping concentration and with increasing temperature. This is a particularly valuable property since it allows for optical temperature sensing via self-referenced luminescence decay time. The temperature sensitivity estimated by a linear fit for temperatures between 0 ${}^{\circ }$C and 80 ${}^{\circ }$C was between −0.5%/K and −0.8%/K, which is significantly higher than for the previously reported other Mn${}^{4+}$-doped emitters and comparable to the sensitive state-of-the-art lifetime-based optical thermometers.

To demonstrate the potential of this material, a fiber optic microsensor was fabricated and tested in comparison to a standard resistance thermometer. The microsensor showed a robust, reproducible and very fast response time, with $t_{90}$ below 1 s in the liquid phase and 1.1 s in the gas phase. An additional advantage of the realized microsensor is its very small dimensions, with a tip diameter of 400 μm, which can be made even smaller by fiber tapering or employing fibers with smaller diameters. Additionally, fiber optic sensors provide the possibility of a contactless read-out, for example through the transparent wall of a reactor.

It can be concluded that Mn${}^{4+}$-activated magnesium titanate represents a highly promising material for optical temperature monitoring at physiological conditions and ambient temperatures due to combination of several attractive features: straightforward synthesis, excellent chemical and photochemical stability of the inorganic phosphor, high temperature coefficients in the range from −50 ${}^{\circ }$C to 150 ${}^{\circ }$C. Additionally, being based on self-referenced decay time read-out, contrary to most rare earth-based systems, which are ratiometric, it does not need a more complex (i.e., more bulky and expensive) optical set-up with two emission channels. Excitability with bright blue LEDs and large Stokes shift facilitate construction of compact and low cost read-out systems.

## Figures and Tables

**Table 1:** Quantum yield and luminescence decay times for various Mn${}^{4+}$ concentrations at 20 ${}^{\circ }$C.

Mn${}^{4+}$ Concentration (mol%)	Quantum Yield (%)	Decay Time (ms)
0.05	87	0.50
0.10	87	0.49
0.40	36	0.44
1.00	15	0.35

## Abbreviations

The following abbreviations are used in this manuscript:

LOF	Lab-on-Fiber
LED	light-emitting diode
TCSPC	time-correlated single photon counting
IRF	instrument response function
PT-100	platinum resistance thermometer
OD	optical density
